# The effect of blister packaging Iron and Folate on adherence to medication and hemoglobin levels among pregnant women at National Referral Hospital antenatal clinics in a low to middle income country: a Randomised Controlled Trial (The IFAd Trial)

**DOI:** 10.1186/s12884-022-04507-3

**Published:** 2022-03-03

**Authors:** Josaphat Byamugisha, Nancy Adero, Tusuubira S. Kiwanuka, Christine K. Nalwadda, Peter Ntuyo, Imelda Namagembe, Evelyn Nabunya, Emily Nakirijja, Robert Mwadime-Ngolo, David Christopher Mukasa, Sam Ononge

**Affiliations:** 1 Department of Obstetrics and Gynecology, College of Health Sciences Makerere University, P.O Box 7072, Kampala, Uganda; 2 JSI Research & Training Institute Inc., Boston, USA; 3 Baylor College of Medicine COE, Kampala, Uganda; 4 Mengo Hospital, Kampala, Uganda; 5 School of Public Health, College of Health Sciences, Makerere University, Kampala, Uganda; 6 Directorate of Obstetrics and Gynecology, Mulago National Referral Hospital, Kampala, Uganda; 7 University Hospital, Makerere University, Kampala, Uganda

**Keywords:** IFA, Blister, Loose packaging, Adherence, Pregnant women

## Abstract

**Introduction:**

Anemia in pregnancy is an important global public health problem. It is estimated that 38% of pregnant women worldwide are anemic. In Africa, literature from observational studies show 20% of maternal deaths are attributed to anemia. In Uganda, 50% of pregnant women have iron deficiency anaemia. The proportion of pregnant women receiving Iron-Folic acid (IFA) supplementation has improved. However, the number of IFA pills consumed is still low. We carried out a randomized controlled trial to determine the effect of dispensing blister and loose packaged IFA pills on adherence measured by count on next return visit and hemoglobin levels among pregnant women at two National Referral Hospitals in Kampala, Uganda.

**Methods:**

This trial was conducted between April and October 2016. Nine hundred fifty pregnant women at ≤28 weeks were randomized to either the blister (intervention arm) or loose (control arm) packaged IFA. The participants completed the baseline measurements and received 30 pills of IFA at enrolment to swallow one pill per day. We assessed adherence by pill count and measured hemoglobin at four and 8 weeks. The results were presented using both intention-to-treat and per-protocol analysis.

**Results:**

There were 474 participants in the control and 478 in the intervention arms. Adherence to IFA intake was similar in the two groups at 4th week (40.6 and 39.0%, *p* = 0.624) and 8th week (51.9 and 46.8%, *p* = 0.119). The mean hemoglobin level at 4 weeks was higher in the blister than in the loose packaging arms (11.9 + 1.1 g/dl and 11.8 + 1.3 g/dl, respectively; *p* = 0.02), however, similar at week 8 (12.1 + 1.2 and 12.0 + 1.3, respectively; *p* = 0.23). However, over the 8-week period blister packaging arm had a higher change in hemoglobin level compared to loose package (blister package 0.6 ± 1.0; loose packaging 0.2 ± 1.1; difference: 0.4 g/dL (95% CI: 0.24–0.51 g/dL); *p* = 0.001. There were no serious adverse events.

**Conclusions:**

Our results showed no effect of blister packaging on IFA adherence among pregnant women. However, our findings showed that blister packaged group had a higher hemoglobin increase compared to loose iron group.

**Trial registration:**

No. PACTR201707002436264 (20 /07/ 2017).

**Supplementary Information:**

The online version contains supplementary material available at 10.1186/s12884-022-04507-3.

## Introduction

Anemia in pregnancy is an important global public health problem [1]. It is estimated that 38% of pregnant women worldwide are anemic [1, 2]. In Africa, observational studies suggest that 20% of maternal deaths have been attributed to anemia. In Uganda, a low to middle income country, 50% of pregnant women have iron deficiency anemia and 30% of maternal death are attributed to anemia [3].

Women often become anemic during pregnancy because of the increased demand for iron and other micronutrients [4]. The inability to meet the required level of these substances either as a result of dietary deficiencies or infection results into anemia [5]. In pregnancy, anemia has a significant impact on the health of the fetus and that of the mother. Folic acid deficiency around conception period is associated with neural tubal defects and other non-genetic congenital abnormalities (cleft lip and heart disease). It has been suggested that folic acid deficiency causes placental vasculopathy that contributes to spontaneous abortion, premature placenta abruption and preeclampsia [6, 7]. Similarly, iron deficiency has been observed to decrease the weight of the newborn, increased premature delivery and other complications during delivery. Iron is necessary for the placenta, uterine enlargement, red blood cell synthesis and fetal growth [7, 8].

The World Health Organization (WHO) recommends iron and folic acid supplementation throughout the duration of the pregnancy. WHO and Uganda’s National Anemia Policy recommend that pregnant women should receive a standard dose of 60 mg iron + 400 μg folic acid for at least 6 months; a total of (at minimum) 180 IFA tablets [1, 3].

This policy recommendation is to prevent anemia and iron deficiency (not therapeutically), and 180 tablets would be needed from the start of the second pregnancy trimester to deliveryThe proportion of women receiving iron supplementation improved steadily over the past decade. The progress in the number of IFA pills consumed, however, has been slow [9]. Studies have shown that the use of blister packs containing drugs significantly increased patients’ compliance, compared with traditional means of dispensing drugs in a paper envelope [10, 11].

No trials have been done to assess the effectiveness of blister packaging of Iron-Folic Acid (IFA) on adherence to treatment in low to middle income countries like Uganda. In this trial, we tested the hypothesis that dispensing blister packaged IFA to pregnant women in ANC increases the adherence compared to the loose pills.

## Materials and Methods

### Study Design

We carried out a randomised controlled open label/ non blinded trial to test the effectiveness of blister packaging, and loose packaging of IFA pills on adherence to the supplementation regimen.

### Participants

We included all consenting pregnant women eligible for IFA supplementation in their second trimester up to 28 weeks. We excluded pregnant women with complications like congestive cardiac failure and sickle cell anemia because they needed urgent investigations, intervention and treatment. The study was initially conducted at Mulago National Referral Hospital and later shifted to Kawempe National Referral Hospital antenatal clinics in Kampala, Uganda. Mulago and Kawempe serve as the teaching hospitals for Makerere University College of Health Sciences. The shift from the former to the latter was necessary because the original site was closed to patients for renovation which the study had not anticipated. The pregnant women were advised to seek alternative health facilities for continuation of care. However, majority of these women opted to receive care from Kawempe National Referral Hospital. The antenatal clinics were conducted 5 days a week and an average of 300 pregnant women were reviewed per week. Approximately two thirds of these pregnant women start ANC when the pregnancy is less than 28 weeks. In addition to the routine antenatal services, antenatal clinics provide health education, immunization and investigations including HIV counselling and testing. During the study period i.e. April to October 2016, antenatal clinics were supplying IFA pills in a loose packaging, the antimalarial (Intermittent presumptive treatment) Fansidar, and anti-helminth drug; Mebendazole to all pregnant women.

### Intervention

The pregnant women in the intervention arm received IFA that was packaged in blisters and these were purchased from Uganda Joint Medical Store (https://www.jms.co.ug/). The control group received the IFA in loose form packed in drug envelopes. The IFA in loose packs were identical to blister packaged IFA in colour and taste. The participants in both arms were given thirty pills of IFA and they were instructed to swallow one per day. The quantity dispensed were adequate to last up to the next scheduled visit when she would get a refill. They received the routine antenatal services provided at the facility.

### Procedure

After the health education of the pregnant women by the midwives on each clinic day, the study briefly introduced the objectives and design of the group sessions. We repeated the sessions about the study in every antenatal clinic during the study period. Those interested and willing to join the study were screened for eligibility according to inclusion criteria. The eligible participants were taken through a written informed consent. Those who consented, completed the baseline assessment that included the sociodemographic, medical and obstetric characteristics, history of malarial infection during pregnancy confirmed by laboratory test (microscopy or rapid diagnostic test), and hemoglobin check. The hemoglobin level was determined using a CBC analyzer machine model MEK-6500 K. All study participants were counselled on the importance of IFA, the dose, frequency to be taken, and management of side effects. Participants were advised to report at the next visit with the remaining pills of IFA. Two follow-up visits were scheduled at 4 and 8 weeks from enrolment respectively. During the first visit (4 weeks), pill count was conducted by the research assistants, any adverse effects were documented and blood was drawn for hemoglobin check. The participants were reminded of the importance of taking IFA and another 30 pills were dispensed to them to swallow as stated above. The participants were given appointment to return for the second visits after another 4 weeks. The second visit activities were the same as those in first. The study team made phone calls to participants who had missed their appointment by 1 week. Participants were defined as lost-to-follow-up when we were unable to physically contact them 2 weeks after the expected date of the second appointment.

### Outcome measures

The primary outcome of the study was adherence to the IFA supplementation regimen, which was assessed verbally and using a balance from the 30-day pill count computed at 4 and 8 weeks’ periods. Adherence at return visit was measured by pill count. For participants who reported at scheduled 4 weeks visit, the balance of pills was counted if they had the drug package with them or they were asked the balance of pill left at home. If the count or balance was 2 pills, that indicated that they had swallowed 28 pills as instructed (that is one pill per day), and they were considered to have 100% adherence. Those lost-to-follow-up were considered non-adherent participants. The same assessment was applied at 8th week of second return. We also computed 90% adherence based on pill count (had balance of only 3 tablets on return visit). The secondary outcome was change in hemoglobin level from baseline to 8 weeks.

### Sample size

Based on the study done in Ethiopia that showed adherence among women to iron folate supplementation was 37.2% [12], and our hypothesis that blister packaging of iron folate pills increases compliance by 9 percentage points, with a two sided 5% significance level and 80% power, we calculated a sample size of 481 in each group given an anticipated 10% loss to follow up.

### Randomization

Using a blocked randomisation sampling technique, computer random numbers in blocks of six were generated by a biostatistician. The biostatistician produced an allocation list for the study arms. The allocation sequence was concealed from the research team enrolling and screening participants in serially numbered sealed opaque envelopes (concealed allocation) containing the randomization group. After the research assistant had obtained consent, she would open the next envelope to determine the group assignment, only after the participant was enrolled, completed all the baseline assessment and it was time to allocate the intervention. Because of the nature of the study, it was difficult to blind the implementation of the allocation and measurement of the outcome. The participants recruited were assigned to receive either an appropriately labelled blister pack containing IFA pills or to receiving the standard of practice of the loose packs.

### Data Analysis

Data was entered into a computer using the Epi-Data software package 3.1. Descriptive statistics were used to examine socio-demographic characteristics, medication received, and brief diet history. Univariate analysis was performed using chi-square for categorical variables. The primary outcome was adherence to IFA at the end of 4th and 8th weeks. We compared 100% adherence to IFA between the two group using chi-square. We also performed the comparison at 90% adherence to IFA. For the secondary outcome: we compared hemoglobin change from enrolment to 8th week between the two groups using the independent t-test. We performed both intention-to-treat and per-protocol analysis. Participants who were lost to follow up at both first and second visits were considered none-adherent to IFA supplementation. For other variables with missing data principle of multiple imputation was applied. Literature currently considers multiple imputation for incomplete variables as a state-of-the-art technique because it improves accuracy and statistical power relative to other missing data techniques [13]. Multiple imputation of hemoglobin levels at 4 and 8 weeks was performed using chained equation with five iterations. Hemoglobin level at 4 and 8 weeks were included as predictors in the chained equation along with maternal age, baseline hemoglobin, parity, gestation age and HIV status. Comparison of mean hemoglobin at 4th and 8th week and, average change in hemoglobin between baseline and 8th week was performed on the multiple imputed data set using t test.

### Quality control

The questionnaires were pretested to ensure clarity and consistency. The research assistants were trained prior to the trial. The questionnaires were reviewed daily to ensure data completeness. The research team ensured that active ingredients in both the blister packaged IFA and the loose packs were similar. The only difference was the blister packaging.

### Ethical considerations

Approval to conduct the research was obtained from Mulago Hospital Research and Ethics Committee (MHREC 837) and the Uganda National Council for Science and Technology (Ref No. HS1897). Informed written consent was obtained from the participants. We informed the participants about the risks involved in this research such as pain from the prick when withdrawing the blood samples, with an explanation about the purpose, procedure of the research and the projected use of the data, with an assurance of confidentiality. It was emphasized that their participation was voluntary and that they could withdraw from the study at their will. Their withdrawal would not affect the care they received at the hospital.

## Results

**Fig. 1:**
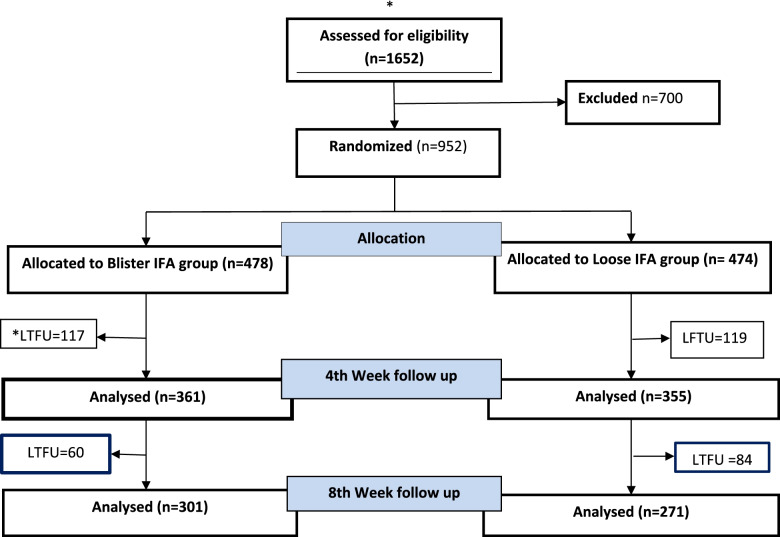
IFAd Trial profile as per CONSORT guidelines. *LTFU: lost to follow up

The two hospitals contributed participants to the study. One thousand six hundred fifty-two (1652) women who came for antenatal care during study period were screen for eligibility. A total of 950 participants gave informed consent and were recruited into the trial, 474 (in the IFA pills in loose packaging arm and 478 in the IFA in blister packaging arm. A total of 380 (39.9%) women were lost to follow up; 42.8% in the control arm and 37.3% in the intervention arm. Reasons for lost-to-follow-up included the closure of the primary hospital where the recruitment started and the pregnant women had to relocate to alternate health facilities near their residence for continued care. The study tried to contact the women through phone calls, however, the study team were unable to reach them as phone contacts were switched off or because women could not be traced by the names and phone numbers they had given the study team. Figure 1 shows the flow of study participants according to the Consort guidelines for randomized trials.

### Baseline characteristics

Table 1 shows the baseline characteristics of the study population. The age range was 15 to 43 years. The two arms did not differ regarding mean haemoglobin levels, socio-demographic characteristics, medical and dietary history. One third of the study population were primigravidae, with a similar proportion in each arm diagnosed and treated for anemia (28.8% in loose IFA and 26.2 in blister IFA arms respectively).

**Table 1:** Baseline characteristics of the study participants

Variable	IFA in blister packaging	IFA in loose packaging	All
Number of participants [n (%)]	476 (50.1)	474 (49.9)	950
Age [mean ± SD]	25.7 ± (5.3)	25.2 ± 4.8	
Primigravida [n (%)]	159 (33.4)	164 (34.7)	323 (34.0)
Gestation age in weeks [mean ± SD]	21.9 ± 3.3	23.3 ± 3.4	
Married [n (%)]	385 (80.9)	390 (82.3)	775 (81.6)
Education			
No education [n (%)]	5 (1.1)	4 (0.8)	9 (1,0)
Primary school (7 years) [n (%)]	96 (20.2)	97 (20.5)	193 (20.3)
Secondary school (6 years) [n (%)]	286 (60.1)	311 (65.6)	597 (62.8)
Post-secondary (3–5 years) [n (%)]	89 (18.7)	62 (13.1)	151 (16.0)
Unemployed [n (%)]	223 (46.8)	206 (43.5)	429 (45.1)
Medical history			
HIV positive [n (%)]	36 (7.6)	42 (8.9)	78 (8.3)
P falciparum infection pregnancy [n (%)]	125 (26.4)	133 (28.2)	258 (27.3)
Taken IFA before enrolment [n (%)]	172 (36.1)	179 (37.8)	351 (36.9)
IPT doses			
None [n (%)]	329 (69.1)	332 (70.0)	661 (69.6)
One [n (%)]	130 (27.3)	130 (27.4)	260 (27.4)
Two [n (%)]	17 (3.6)	12 (2.5)	29 (3.0)
Deworming in pregnancy			
None [n (%)]	379 (79.6)	380 (80.2)	759 (79.9)
At least one dose [n (%)]	97 (20.4)	94 (19.8)	191 (20.1)
Hemoglobin [mean ± SD]	11.6 ± 1.2	11.6 ± 1.4	
Anemia categories			
No anemia [n (%)]	347 (72.9)	333 (71.2)	680 (71.6)
Any anemia < 11.0 mg/dl [n (%)]	129 (27.1)	141 (29.8)	270 (28.4)
Dietary history			
Meals per day [mean ± SD]	2.6 ± 0.9	2.5 ± 0.8	
Fruits per week [mean ± SD]	4.0 ± 2.9	3.8 ± 2.6	
Green veg. Per week [mean ± SD]	3.0 ± 2.4	2.9 ± 2.5	
Meat per week [mean ± SD]	1.8 ± 1.7	1.7 ± 1.8	
Fish per week [mean ± SD]	1.5 ± 1.8	1.6 ± 1.7	
Chicken per week [mean ± SD]	0.6 ± 1.0	0.7 ± 1.3	
Beans/peas per week [mean ± SD]	3.8 ± 2.5	4.0 ± 2.3	

*SD* Standard Deviation

#### Follow-up assessment at 4th and 8th weeks

More than 90% of the women in both arms rated their understanding of the information provided during counselling to be above 4 on a five-point Likert scale, at week 4 and 8.

##### Adherence to IFA regimen

Adherence was analysed among the mothers who returned within 30 days of either enrolment at 4th and 8th week follow up respectively (Table 2 and Table 3). There was no difference between the two arms in the proportion of mothers who returned within 30 days. Based on the pill count, the adherence was similar in the two groups at 4 and 8 weeks, respectively. Even when the adherence criteria was relaxed from 100% (mothers consumed 28 out of the 30 pills that were given) to 90% (mothers consumed 25 out of the 30 pills that were given) the level of adherence was similar in the two groups (Table 2).

**Table 2:** Adherence to IFA at first visit (4th week) and second visit (8th week) analysed by intention to treat

Characteristics	IFA in blister packaging *n* = 478	IFA in loose packaging *n* = 474
First follow up visit or return visit (week 4)		
Participants who returned for first follow up visit	361	355
Participants who returned within 30 days [n (%)]	250 (52.3)	242 (51.1)
Balance of pills counted on first return visit [mean ± SD]	3.5 ± 3.9	3.5 ± 4.6
100% adherence among participants on first return visit [n (%)]	194 (40.6)	185 (39.0)
90% adherence among participants on first return visit [n (%)]	220 (46.4)	220 (46.0)
Second follow up visit (week 8)		
Participants who returned for followed up at second visit (8 week)	301 (60.3)	271 (55.3)
Balance of pill count on second return visit [mean ± SD]	2.6 ± 3.3	2.9 ± 3.7
100% adherence among participants on second return visit [n (%)]	248 (51.9)	222 (46.8)
90% adherence among participants returning on second visit [n (%)]	286 (59.8)	268 (56.5)

**Table 3:** Adherence to IFA at first visit (4th week) and second visit (8th week) analysed per protocol

Characteristics	IFA in blister packaging	IFA in loose packaging
First Follow up visit or return visit (week 4)		
Participants who returned for first follow up visit	361	355
100% adherence among participants on 1st return visit [n (%)]	194 (53.7)	185 (52.1)
Second follow up visit (week 8)		
Participants who returned on second (8 week) follow up visit	302 (60.3)	271 (55.3)
Balance of pill count on returned visit [mean ± SD]	2.2 ± 2.8	2.6 ± 3.2
100% adherence on second return visit [n (%)]	199 (66.0)	159 (58.7)

##### Hemoglobin concentration

The mean hemoglobin level at 4 weeks was higher in the blister than in the loose packaging arms (11.9 + 1.1 g/dl and 11.8 + 1.3 g/dl, respectively; *p* = 0.02). But the hemoglobin levels were similar at week 8 (12.1 + 1.2 and 12.0 + 1.3, respectively; *p* = 0.23). However, over the 8-week period of the trial, participants that were on blister packaging arm had a higher change in hemoglobin level compared to those on loose package (blister package 0.6 ± 1.0; loose packaging 0.2 ± 1.1; difference: 0.4 g/dL (95% CI: 0.24–0.51 g/dL); *p* = 0.001. There were no serious adverse events reported in this study.

## Discussion

We carried out the first ever randomized controlled trial, in a low to middle income country, to assess the impact of IFA pills provided in blister packaging compared to loose packs on adherence to medication and hemoglobin concentration. Our findings showed that 8 weeks adherence to iron among pregnant women attending the antenatal clinic in two large tertiary care centers in Uganda was similar between blister and loose packaged supplements. However, the mean increase in hemoglobin level was higher among the blister packs. There are many factors that can affect the adherence to IFA supplementation. The level of education of the mothers has a positive bearing on adherence to IFA supplementation. Studies show that the risk of non-adherence is very high when patients cannot read and understand basic written medical instructions. Patients’ health beliefs are affected by their health literacy, and these beliefs are also contributors to non-adherence. Patients’ attitudes and group norms influence adherence. A systematic review and meta-analysis mentioned educational status of the mothers as one of the key determinants of adherence to IFA supplementation [14]. Another important factor influencing adherence is patients’ ability to remember the details of the recommendations made to them. Studies have repeatedly shown that forgetting to take (or how to take) medications are a major contributor to non-adherence [15] Another major area is interpersonal dynamics between the health care provider and the beneficiary. Patients who feel that their physicians communicate well with them and actively encourage them to be involved in their own care tend to be more motivated to adhere [16, 17]. Successful communication between physicians and patients promotes greater patient satisfaction with medical care, which in turn fosters higher levels of adherence. Related to that area is the opportunity for participatory decision making. Studies have found that both patient satisfaction and patient adherence are enhanced by patients’ involvement and participation in their care [16, 18]. Physician–patient partnership and social support from health professionals, as well as from members of the patient’s social network, are essential to patients’ adherence to recommended treatments [19, 20].

Adherence to a drug regimen requires a coordinated effort from health care providers, patients/beneficiaries, and a network of peers, in addition to broader issues related to healthcare access and equity. Based on the input of the health service providers and the mothers, it appears that adherence to IFA supplementation regimen is influenced by knowledge of benefits of IFA rather than blister packaging as demonstrated in this trial. In one of the micronutrient publications, knowledge did not come out as a main issue [21]. Barriers to IFA supplementation included low priority for IFA within maternal health programs, inadequate supplies, low utilization and weak demand, lack of convincing evidence of effectiveness, missing community-based delivery platforms to complement the ANC and insufficient bundling of interventions to address the multiple causes of anemia [21]. In another study done in Vietnam, socioeconomic status, ethnicity, occupation (farmer) and parity were noted to be the key determinants of adherence [22].

However, in another study done among pregnant women in Eritrean refugee camps, low level of knowledge about anemia in pregnancy amongst mothers, fear of side effects, occurrence of side effects forgetfulness, and inadequate IFA in health facilities were among the major dimensions associated with discontinuation among Iron Folic Acid end users [23]. A study done in Kiambu County, Kenya recommends counselling of mothers about management of side effects of IFA supplements as a way of increasing adherence [24]. There is need to prioritize programs on IFA supplementation in pregnancy. A study conducted across 22 countries with high burden of under nutrition revealed that many of them did not have national guidelines on IFA supplementation in antenatal care [25].

Although, the study was not powered to determine the effectiveness of blistering on hemoglobin change, our findings showed that participants in the intervention arm had higher hemoglobin increase than those in loose package. However, as shown in Table 2, adherence was higher among blister package group than among the loose package (52% vs 47%). This may have contributed to a higher mean hemoglobin in blister group than loose packaged group.

There were a few considerations before generalizing the results from our trial. There was no difference in the non-response among the two groups. Additionally, Mulago and Kawempe are tertiary care hospitals, with good access to essential medicines like IFA.

### Strengths of the trial

The strengths of the study include the randomized trial design that gave each pregnant woman equal chance to be in intervention or control arms and the study allowed us to assess the intervention in a real life setting in a Low to Middle Income Country. The research team managed to access a similar research site when the initial one was closed for renovation.

### Limitations of the trial

It was not possible to mask the participants or the research team, to the intervention given its nature. Secondly, loss to follow-up of 39.8% was high, however, characteristics of women at enrollment were not different from those followed up (data not shown), and similar in the control and intervention groups. The study setting changed in the course of the trial. The antenatal patients were shifted from Mulago National Referral Hospital to Kawempe National Referral Hospital to allow room for renovations and remodelling of the former. The two hospitals are about 5 Kilometres (3 miles) apart. This could have led to loss-to-follow-up of some of the participants. However, the catchment population is quite similar and is urban and peri-urban.

## Conclusion

Our results showed no effect of blister packaging on IFA adherence among pregnant women. Changes in hemoglobin did not differ in the two groups. However, our findings showed that blister packaged group had a higher hemoglobin increase compared to loose iron group.

## Supplementary Information

**Additional file 1.**

## Data Availability

The data sets used and analyzed during the study are included in the supplementary information files.
